# Retraction Note: MicroRNA-325-3p inhibits cell proliferation and induces apoptosis in hepatitis B virusrelated hepatocellular carcinoma by downregulation of aquaporin 5

**DOI:** 10.1186/s11658-023-00499-w

**Published:** 2023-10-20

**Authors:** Zhitao Zhang, Yanzhen Han, Guangxin Sun, Xiaohong Liu, Xiaoyan Jia, Xiangjun Yu

**Affiliations:** 1 General Surgery V Ward, Affiliated Hospital of Hebei Engineering University, Handan, 056002 Hebei People’s Republic of China; 2 Clinical Laboratory, Handan Infectious Disease Hospital, Handan, 056002 Hebei People’s Republic of China

**Retraction Note : Cellular & Molecular Biology Letters (2019) 24:13** 10.1186/s11658-019-0137-1

The Editor in Chief has retracted this article because of concerns about the data presented. Specifically, the flow cytometry plots presented in Fig. 1E show a significant similarity to the plots presented in a previously published paper with no common authors [1]. Additionally, the ACP5 blots presented in Fig. 2F appear highly similar to two blots presented in a paper published around the same time as this paper with no common authors [2]. The Editor in Chief no longer has confidence in the reliability of the data. All authors agree to this retraction.
